# A VCII-Based Stray Insensitive Analog Interface for Differential Capacitance Sensors

**DOI:** 10.3390/s19163545

**Published:** 2019-08-14

**Authors:** Gianluca Barile, Leila Safari, Giuseppe Ferri, Vincenzo Stornelli

**Affiliations:** Department of Industrial and Information Engineering and Economics, University of L’Aquila, 67100 L’Aquila, Italy

**Keywords:** differential capacitive sensor, readout circuit, high sensitivity, VCII, parasitic insensitive

## Abstract

In this paper, a novel approach to implement a stray insensitive CMOS interface for differential capacitive sensors is presented. The proposed circuit employs, for the first time, second-generation voltage conveyors (VCIIs) and produces an output voltage proportional to differential capacitor changes. Using VCIIs as active devices inherently allows the circuit to process the signal in the current domain, and hence, to benefit from its intrinsic advantages, such as high speed and simple implementation, while still being able to natively interface with voltage mode signal processing stages at necessity. The insensitiveness to the effects of parasitic capacitances is achieved through a simple feedback loop. In addition, the proposed circuit shows a very simple and switch-free structure (which can be used for both linear and hyperbolic sensors), improving its accuracy. The readout circuit was designed in a standard 0.35 μm CMOS technology under a supply voltage of ±1.65 V. Before the integrated circuit fabrication, to produce tangible proof of the effectiveness of the proposed architecture, a discrete version of the circuit was also prototyped using AD844 and LF411 to implement a discrete VCII. The achieved measurement results are in good agreement with theory and simulations, showing a constant sensitivity up to 412 mV/pF, a maximum linearity error of 1.9%FS, and acknowledging a good behavior with low baseline capacitive sensors (10 pF in the proposed measurements). A final table is also given to summarize the key specs of the proposed work comparing them to the available literature.

## 1. Introduction

In recent years, capacitive sensors have found numerous applications in measuring pressure, displacement, velocity, etc. [1,2,3,4,5,6,7,8,9,10]. Inside this category, there are the differential capacitive sensors, which exhibit robust performance against unwanted common-mode inputs and are capable of detecting low values of capacitive variations. Thanks to the recent advancements in CMOS technology like silicon micromachining, capacitive sensors can be easily implemented through this technology. Therefore, it is viable to integrate the sensor itself and the readout circuitry on a single chip, advancing towards the interesting field of smart sensors. The readout circuit of a smart sensor requires demanding specifications such as easy integration; low-voltage, low-power operation; high accuracy; and high speed. As the noise performance of the readout circuitry limits the overall precision, it must feature low input and output noise. In addition, the ever-growing need of markets for portable devices makes low power consumption an important factor. Therefore, as the performance of complete smart sensor is highly dependent on the readout circuitry, the development of an interface which can fulfil the above-mentioned requirements has become a hot research topic of our times.

A survey in literature reveals that different approaches have been adopted to design readout circuits for differential capacitors. However, most of the circuits presented so far fail to fulfil important features such as easy integration, low power consumption, and high accuracy. For example, the circuit presented in [11,12] includes several high value resistors, capacitors, and a large number of active building blocks, which results in large chip area and very high power consumption. The solutions reported in [13,14,15,16,17,18,19] employ a large number of switches and require additional controlling clock signals. Moreover, the clock feedthrough and charge injection errors of switches limit their maximum achievable accuracy. The readout circuit proposed in [8,20,21] operates on a bridge-based approach. Its main drawback is its complicated structure, which includes a differential amplifier using several matched resistors. Although adopting current mode signal processing leads to circuits with reduced complexity, high speed, and low voltage operation [7,8,10,22], when the sensor is excited by a current source, the parasitic capacitances become a limiting factor since it consumes part of the input current. As a consequence, the readout circuitry is applicable in those cases where the sensor capacitance is much larger than parasitic capacitance. For example, the interfaces presented in [7,22] are based on the current mode approach, enjoying a very simple structure, but the circuits allow less than 40% of capacitor changes and suffer from a limited accuracy. In the current mode readout circuit presented in [10], a mechanism has been adopted to compensate the effect of parasitic capacitance. However, there are three switches in the circuitry, which demands a control by additional clock signals. Moreover, the clock feedthrough and charge injection errors limit the circuit’s overall accuracy.

In this paper, we describe and prototype a novel current mode interface based, for the first time, on the second-generation voltage conveyor, a three terminal active block that, differently from Op-Amps or second-generation current conveyors (CCIIs), allows for both current domain and voltage domain signal processing without adding extra components or losing performances [23,24,25,26,27,28,29,30]. The interface fulfils many important features namely: Very simple implementation and insensitivity to large sensor parasitic capacitances thanks to an active compensation of the current taken from them. Notably, even when the parasitic capacitance has a large value in the order of the baseline, the proposed circuit exhibits high accuracy and high linearity with high read time, which are comparable to those of previously reported works, while it employs a very simple structure and avoids using any complicated switching circuitry. As an innovative solution, to charge and discharge the sensor capacitors, a square wave current signal is used to excite the sensor. Therefore, the errors and complexity related to switches are avoided. As a result, the proposed circuit features high measure accuracy and high linearity even at maximum sensor variation. To cancel the effect of parasitic capacitance, the current wasted by the parasitic capacitance is determined by a simple second-generation voltage conveyors (VCII)-based summing circuitry and an appropriate compensation current is fed back to input. Therefore, low value sensor changes are detectable by the readout circuitry. The differential capacitor variation is measured by a simple VCII-based current differencing circuit, which produces an output voltage proportional to sensor variation at a low impedance voltage output port. The circuit is completely suitable for integration as it enjoys a very simple structure employing CMOS transistors, single grounded capacitor, and two low-valued resistors. To consider the effect of nonidealities, and to confirm the effectiveness of the working principle, the circuit has been prototyped and measurement results are given. The outline of the presented work is as follows. In section two the basic theory of differential capacitor sensor is presented. In section three a general overview of the main VCII features is given, together with the detailed description of the proposed interface. In section four the simulation results of the designed readout circuit and the measurements on the prototyped circuit are given. Finally, section five concludes the paper.

## 2. Basic Theory

**Figure 1:**
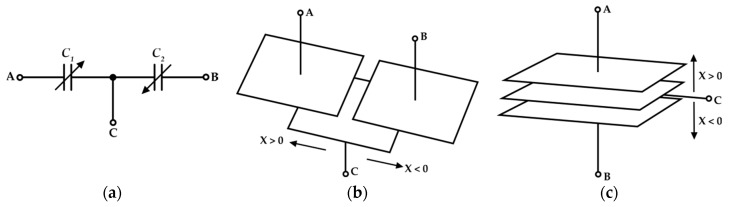
Differential capacitive sensor (**a**) schematic and structure when the measurand influences (**b**) the overlapping area of plates; (**c**) the distance between plates.

Figure 1 shows the schematic and structural diagram of a differential capacitive sensor. As it is shown, a differential capacitive sensor has three plates, two of them fixed and the middle one capable of moving. The parameter *x* is defined as the variation of *C*_1_ and *C*_2_ (under the action of the measurand) with respect to each capacitor value in static condition (*C*_0_/2 = *C_bl_*), called baseline value. In the case when the overlapping area of capacitors changes under the action of a measurand (Figure 1b), there will be a linear relationship between *C*_1_–*C*_2_ and measurand *x*, as it is stated in Equation (1). In the case when the stimulus affects the distance between capacitors plates (Figure 1c), the relationship between *C*_1_–*C*_2_ and the measurand *x* becomes hyperbolic, according to Equation (2).

(1) $$C_{1,2}=C_{bl}\left(1\pm x\right)$$

(2) $$C_{1,2}=\frac{C_{bl}}{1\mp x}.$$

For both linear and hyperbolic cases, the relationship between *C*_1_–*C*_2_ and *x* (−1 < *x* < 1) is a ratiometric parameter expressed by Equation (3):

(3) $$x=\frac{C_{1}-C_{2}}{C_{1}+C_{2}}=\frac{\Delta C}{C_{0}}.$$

**Figure 2:**
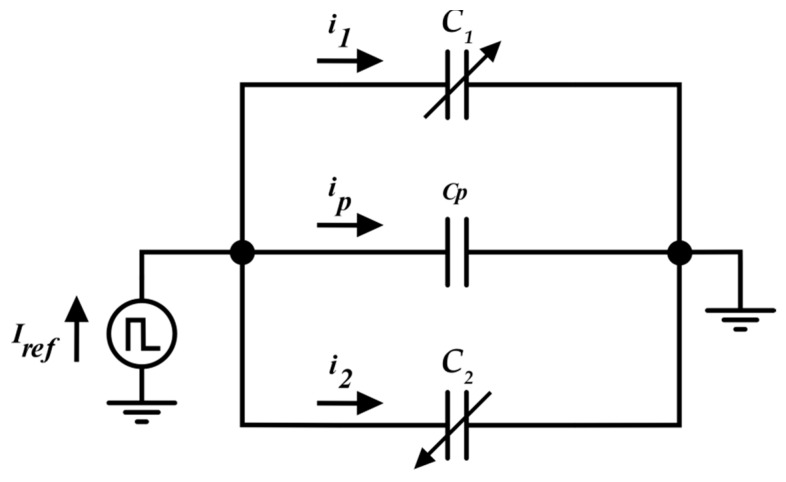
Differential capacitive sensor excited by a current source, where parasitic capacitance *C_p_* is evidenced.

Figure 2 shows a differential capacitor (*C*_1_, *C*_2_), and its parasitic capacitance (*C_p_*), excited by a current source. In the ideal case where there is no parasitic capacitance (*C_p_* = 0), the value of *x* can be obtained by simply writing *i*_1_ − *i*_2_ as:

(4) $$i_{1}-i_{2}=I_{ref}\frac{C_{1}}{C_{1}+C_{2}}-I_{ref}\frac{C_{2}}{C_{1}+C_{2}}=I_{ref}\frac{C_{1}-C_{2}}{C_{1}+C_{2}}=I_{ref}x.$$

In the real case where *C_p_* exists, a part of *I_ref_* (namely, *i_p_*) is taken by *C_p_*. In this case, we can write:

(5) $$i_{p}=I_{ref}-\left(i_{1}+i_{2}\right).$$

Currents *i*_1_ and *i*_2_ can be written as:

(6) $$i_{1}=\lambda \left(I_{ref}-i_{p}\right)\;i_{2}=\left(1-\lambda \right)\left(I_{ref}-i_{p}\right)$$

where the parameters *λ* and (1 − *λ*) can be respectively written, according to the Kirchhoff current law (KCL), as:

(7) $$\lambda =\frac{C_{1}}{C_{1}+C_{2}}\;1-\lambda =\frac{C_{2}}{C_{1}+C_{2}}$$

We can therefore use the difference between the two currents flowing into the sensor to evaluate the *x* parameter:

(8) $$i_{1}-i_{2}=\frac{C_{1}}{C_{1}+C_{2}}\left(I_{ref}-i_{p}\right)-\frac{C_{2}}{C_{1}+C_{2}}\left(I_{ref}-i_{p}\right)=\left(I_{ref}-i_{p}\right)x.$$

As it is seen from Equation (8), the effect of the parasitic capacitance is then a loss of sensitivity of any interface that uses the difference of the two currents in order to evaluate the measurand *x*. This can be negligible for high baseline sensors (thousands of pF), but it drastically reduces the sensitivity of the readout circuit for small baseline sensors (tens of pF or less). In the following section, the proposed approach to cancel the effect of *C_p_* is also presented.

## 3. The Proposed Interface

**Figure 3:**
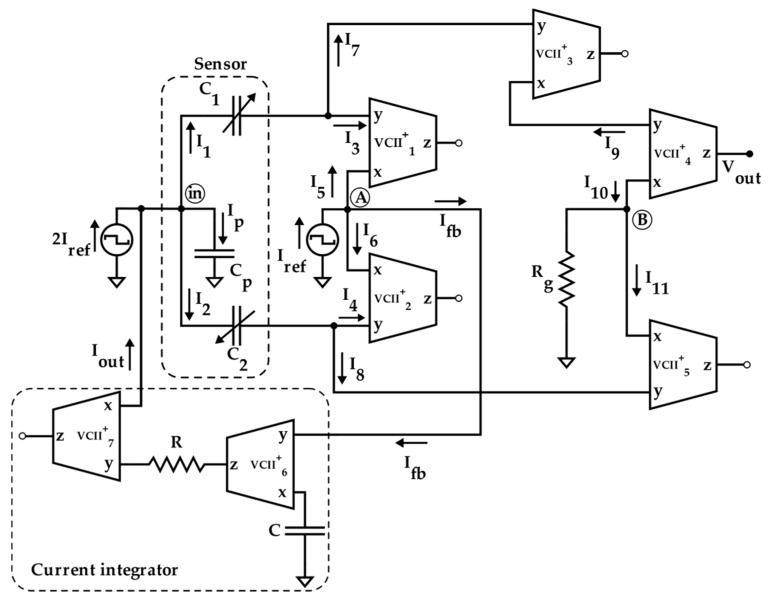
The proposed second-generation voltage conveyors VCII based interface.

The proposed readout circuitry is shown in Figure 3. It utilizes VCII blocks and operates based on a capacitance variation to voltage conversion. VCII can be considered as the dual version of the well-known second-generation current conveyor (CCII). While its introduction is dated back to 2001 [23,24], it is recently gaining more attention [24,25,26]. VCII has been profitably used in fields like current-mode Wheatstone bridges [27], active filters [28], and interfaces for silicon photomultipliers [29,31]. It is characterized by a current buffer between Y and X terminals (having named *β* the ratio between *I_x_* and *I_y_*) and a voltage buffer between X and Z terminals (having named *α* the ratio between *V_z_* and *V_x_)*. The Y and Z terminals are of the low impedance kind (ideally zero). The X node is a high impedance terminal (ideally infinite). The relationship between current and voltage signals of VCII is represented as:

(9) $$I_{x}=\pm \beta I_{y},V_{z}=\alpha V_{x}.$$

In the ideal case *α* and *β* are in unity.

With the aim of cancelling the effect of parasitic capacitance *C_p_*, making the readout insensitive to it, the current taken by *C_p_* is evaluated by subtracting the sum of the currents flowing through the sensor capacitances from the reference current and then feeding it back using a current loop (*I_fb_*). Therefore, its effect on the overall sensitivity is cancelled.

As an innovative approach, the sensor is excited by a square wave current signal. By this solution, the charging and discharging of capacitors are automatically performed without any need to use switches. There are many implementations of square wave current source such as [32], which can be easily integrated together with interface circuit. Here, in order to test the behavior of the interface alone and to eliminate sources of uncertainties, it has preliminarily been implemented by an external voltage source followed by a resistor and a current.

The proposed circuit is composed of three main sections as follows:

1. The first part is a VCII-based current summing/subtracting block composed of VCII_1_–VCII_2_ and a current source equal to *I_ref_*. The sensor input is excited by a current source equal to 2*I_ref_*. The virtual ground and low impedance at Y terminal of VCII keeps the sensor second terminal to ground. In this section, according to Equation (5), the sum of *I*_1_ and *I*_2_ is produced and the result is subtracted from *I_ref_* to obtain *I_fb_*, which, suitably integrated, generates the current taken by *C_p_* (*I_out_* tends to −*I_p_*).
2. The second part is the control section, which feeds *I_fb_* to the sensor input. As it is shown in Figure 3, it is a VCII-based current-input—current-output integrator composed of the two VCII blocks, a resistor, and a grounded capacitor.
3. In the third part (output section), the difference between *I*_1_ and *I*_2_ is produced and converted to a proportional voltage signal. This operation is performed by three VCII blocks (VCII_3_–VCII_5_) and resistor *R_g_*.

The detailed operation of the proposed circuit can be analyzed as follows. At the input node (node in), considering *I_out_* = 0 at the initial time and writing KCL, it results:

(10) $$2I_{ref}=I_{1}+I_{2}+I_{p}.$$

Thanks to the virtual ground at Y ports of VCIIs, using Equation (6), for *I*_1_ and *I*_2_ we have:

(11) $$I_{1}=\lambda \left(2I_{ref}-I_{p}\right)$$

(12) $$I_{2}=\left(1-\lambda \right)\left(2I_{ref}-I_{p}\right).$$

As the used VCIIs have a similar internal structure at Y port, *I*_1_ and *I*_2_ will be split by half. Therefore *I*_3_, *I*_4_, *I*_7_, and *I*_8_ are obtained as:

(13) $$I_{3}=I_{7}=\frac{I_{1}}{2}$$

(14) $$I_{4}=I_{8}=\frac{I_{2}}{2}.$$

Inserting Equations (13) and (14), respectively, into Equations (11) and (12), *I*_3_ and *I*_4_ are given by:

(15) $$I_{3}=\frac{\lambda }{2}\;\left(2I_{ref}-I_{p}\right)$$

(16) $$I_{4}=\frac{1-\lambda }{2}\left(2I_{ref}-I_{p}\right).$$

A simple KCL analysis at node *A* gives:

(17) $$I_{ref}=I_{5}+I_{6}+I_{fb}.$$

By considering the current buffering action between Y and X nodes of VCII, we have:

(18) $$I_{6}\approx \beta_{2}\;I_{4},\approx \beta_{1}\;I_{3}.$$

Using Equation (18), and inserting Equations (15) and (16) into Equation (17), it results:

(19) $$I_{ref}=\beta_{1}\;\frac{\lambda }{2}\left(2I_{ref}-I_{p}\right)+\beta_{2}\;\frac{\left(1-\lambda \right)}{2}\left(2I_{ref}-I_{p}\right)+I_{fb}.$$

By assuming *β*_1_ = *β*_2_ = 1, from Equation (19) we have:

(20) $$I_{fb}=\frac{I_{p}}{2}.$$

In fact, at node *A*, a current comparing action is performed. According to Equation (20), as long as the sum of *I*_5_ and *I*_6_ is not equal to *I_ref_* (i.e., *I*_1_ + *I*_2_ is not equal to 2*I_ref_*), a non-zero compensation current (*I_fb_*) is produced. This current is fed to the input node by the controller circuit, which is a simple VCII-based current integrator as is shown in Figure 3. By assuming virtual ground at Y port of VCII, the relationship between the input and output currents of the integrator can be expressed as:

(21) $$I_{out}=\alpha_{7}\beta_{7}\beta_{8}\frac{1}{sRC}I_{fb}.$$

The design of the integrator (and, in general, of the feedback controller) determines the readout speed of the interface. In particular, its time constant RC must be sufficiently smaller than the half-period of the reference current *I_ref_*, ensuring that the loop reaches the steady state before the reference signal changes polarity, consequently, completing an effective measurement.

To find the output voltage, we assume that *I_p_* is provided by the feedback circuitry. The output voltage is equal to the voltage produced at node *B*, which can be written as:

(22) $$V_{out}=\alpha_{4}V_{B}=\alpha_{4}R_{g}\left(I_{10}-I_{11}\right).$$

For *I*_10_ and *I*_11_ we have:

(23) $$I_{10}=\beta_{4}I_{9}=\beta_{3}\beta_{4}I_{7}=\beta_{3}\beta_{4}\frac{I_{1}}{2}$$

(24) $$I_{11}=\beta_{5}I_{8}=\beta_{5}\frac{I_{2}}{2}.$$

By assuming *α*_4_ = *β*_3_ = *β*_4_ = *β*_5_ = 1, inserting Equations (23) and (24) into Equation (22) gives the output voltage as:

(25) $$V_{out}\approx \frac{R_{g}\left(I_{1}-I_{2}\right)}{2}=R_{g}I_{ref}x.$$

As Equation (25) indicates, supposing *I_ref_* as a square wave current, the output of the interfaces is a square wave voltage, with the amplitude modulated by the sensor variations (regardless of being positive, *C*_1_ > *C*_2_, or negative *C*_2_ > *C*_1_) and the phase indicating whether *C*_1_ is greater than *C*_2_ (phase equal to 0°), or vice versa (phase equal to 180°). The gain can be easily set by *R_g_*, therefore, the circuit enjoys a tunable sensitivity. In order to define this last parameter, it is convenient to consider Δ*C* as reference magnitude and, without losing generality, it is possible to write:

(26) $$S_{\Delta C}^{Vout}=\frac{dV_{out}}{d\Delta C}=\frac{d\left(R_{g}I_{ref}\frac{\Delta C}{C_{0}}\right)}{d\Delta C}=\frac{R_{g}I_{ref}}{C_{0}}\;\left[\frac{V}{F}\right].$$

## 4. Simulation Results and Measurements

**Figure 4:**
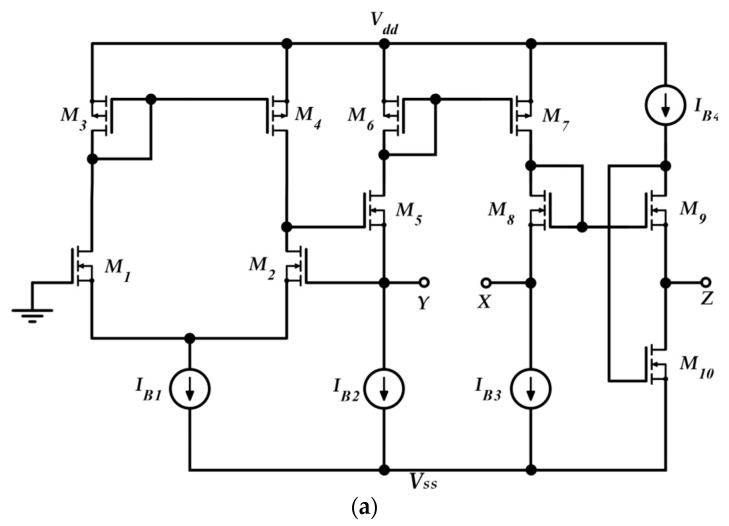

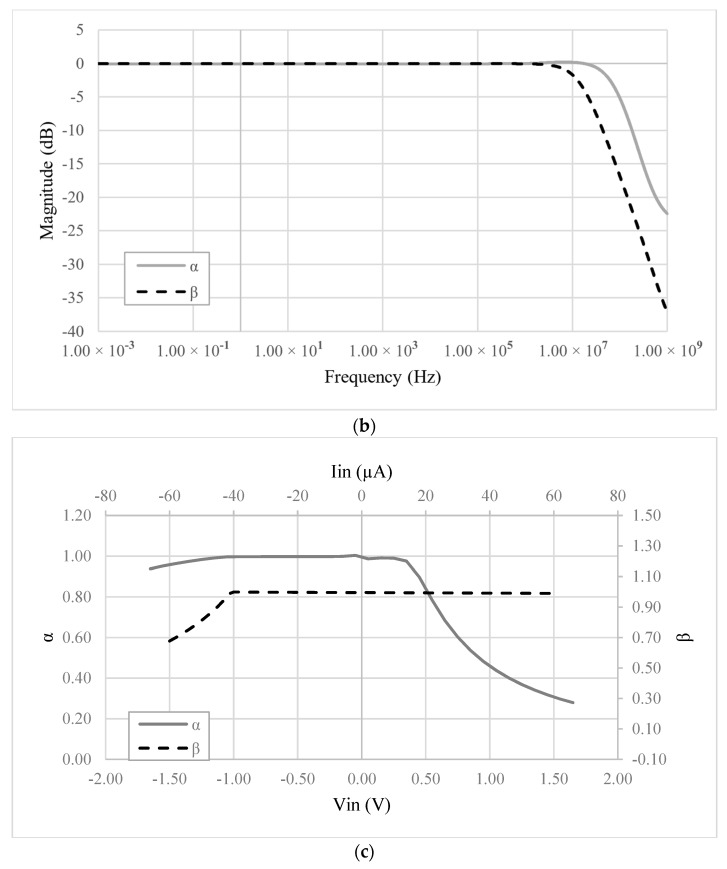
The VCII used in the simulations: (**a**) Topology (see [27]), (**b**) AC performances (determined with a 3 pF load at X), (**c**) DC performances (determined with a 1 kΩ load at Y for β simulations, and with a 100 kΩ load at Z for α simulations).

The proposed readout circuit of Figure 3 has been simulated using Spice in 0.35 μm CMOS technology and a supply voltage of ±1.65 V. For the required VCIIs, the circuit of [27] (Figure 4a) has been used with the same aspect ratios and biasing. Current sources have been implemented by means of simple current mirrors. AC and DC performances of the VCII in terms of *α* and *β* are shown respectively in Figure 4b,c. The low impedance current input (Y) is implemented by a regulated common gate (M_1_–M_5_), while the current transferring action is performed by the current mirror M_6_, M_7_. The flipped voltage follower M_9_, M_10_ allow us to obtain the voltage buffering between the X voltage input and the Z voltage output. The important parameters of the used VCIIs are summarized in Table 1.

**Figure 5:**
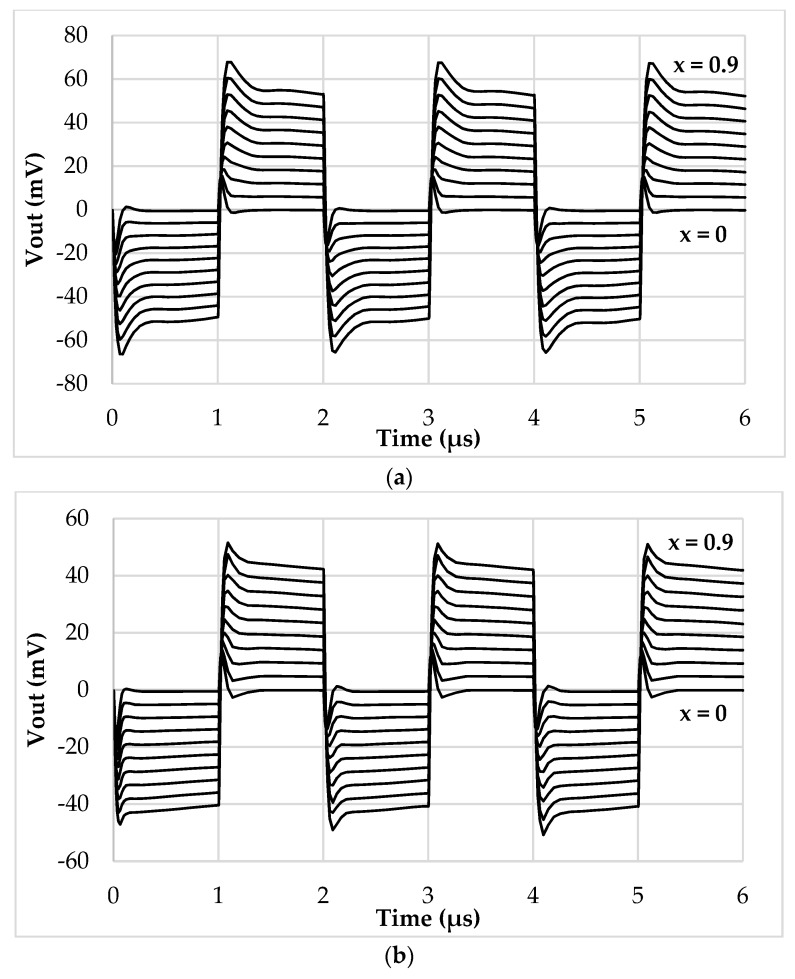
Time domain output voltage with (**a**) compensation enabled and (**b**) compensation disabled.

**Figure 6:**
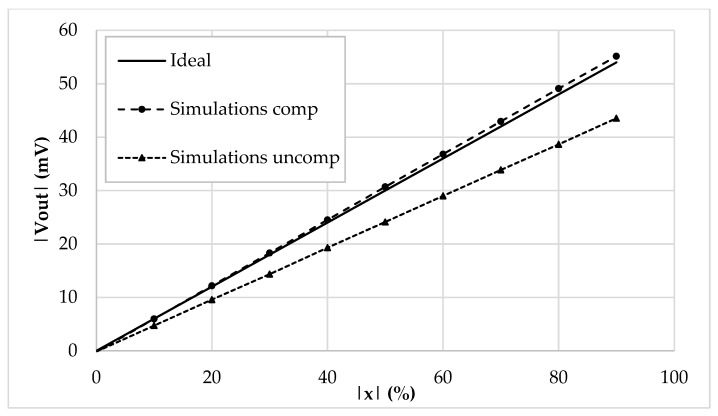
DC characteristic (|*V_out_*| vs. |*x*|) of the integrated interface.

**Figure 7:**
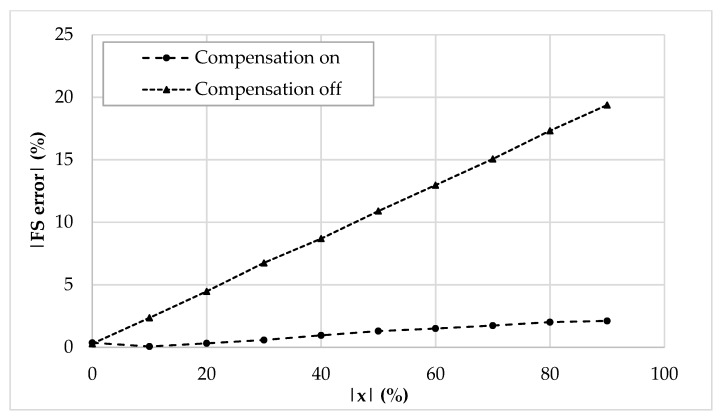
Full-scale error (%) of the compensated and uncompensated integrated interface.

The values of *I_ref_*, *R_g_*, *R,* and *C* have been selected as 5 μA, 12 kΩ, 1 kΩ, and 20 pF, respectively. The frequency of *I_ref_* has been chosen equal to 500 kHz to test the interface behavior close to its maximum operating condition. By setting the sensor baseline *C_bl_* at 10 pF and the parasitic capacitance *C_p_* at 5 pF, the simulation results where the compensation loop has been first enabled, and then disabled, are shown in Figure 5a,b, respectively. For sake of clarity, the picture shows the output voltage only in the case of phase equal to zero. As visible, without the compensation, the amplitude of the output voltage reaches only 80% of full-scale. This behavior is better shown in Figure 6, where the amplitude of each curve has been extracted and compared to ideal values. The full-scale error for each case has also been calculated and is shown in Figure 7: With compensation, the error remains below 2.1%, further proving the effectiveness of the proposed compensation technique. As it is seen, the compensation technique is very effective. It is also observed that thanks to the proposed method, the large parasitic capacitance of 5 pF does not have any effect on the output voltage. In order to analyze both the effects of VCII nonidealities and the effects of mismatches on passive and active components, a mixed simulation was carried out where a Monte Carlo analysis was performed on both PVT conditions for active devices and on tolerances for passive ones. In particular, temperature range was set from −10 °C to 80 °C, supply voltage variation from −5% to 5% of the nominal one, resistors tolerance was set to 5%, while capacitor one to 10%. The overall maximum variation from the nominal reference value was experienced at *x* = 90%, with a deviation from the ideality of about 3 mV. These results are achieved while the circuit consumes 5.2 mW.

**Figure 8:**
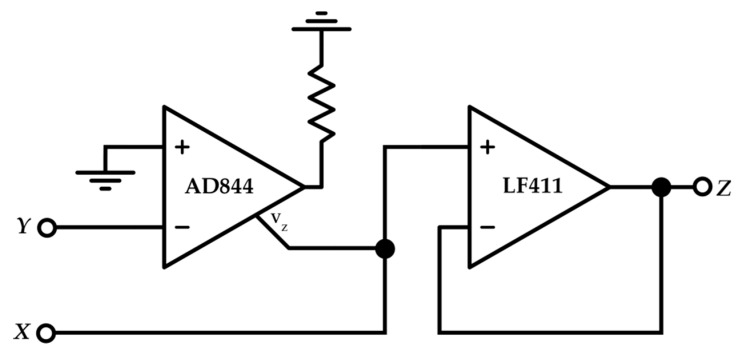
Discrete VCII implementation.

To demonstrate the validity of the proposed architecture before the chip fabrication, and to obtain tangible results, a discrete version of the interface has been simulated and fabricated. The discrete VCII has been implemented as shown in Figure 8, where the current I/O of the AD844 has been used to implement a current buffer, and a simple LF411 has been suitably added to perform a voltage buffering action. Although directly available from the AD844, an external voltage buffer has been adopted in order to gain the possibility of setting an α value greater than unity. For the simulations, taking Figure 3 as reference, *R* and *C* have been again set to 1 kΩ and 20 pF, respectively, while *R_g_* has been set to 33 kΩ.

**Figure 9:**
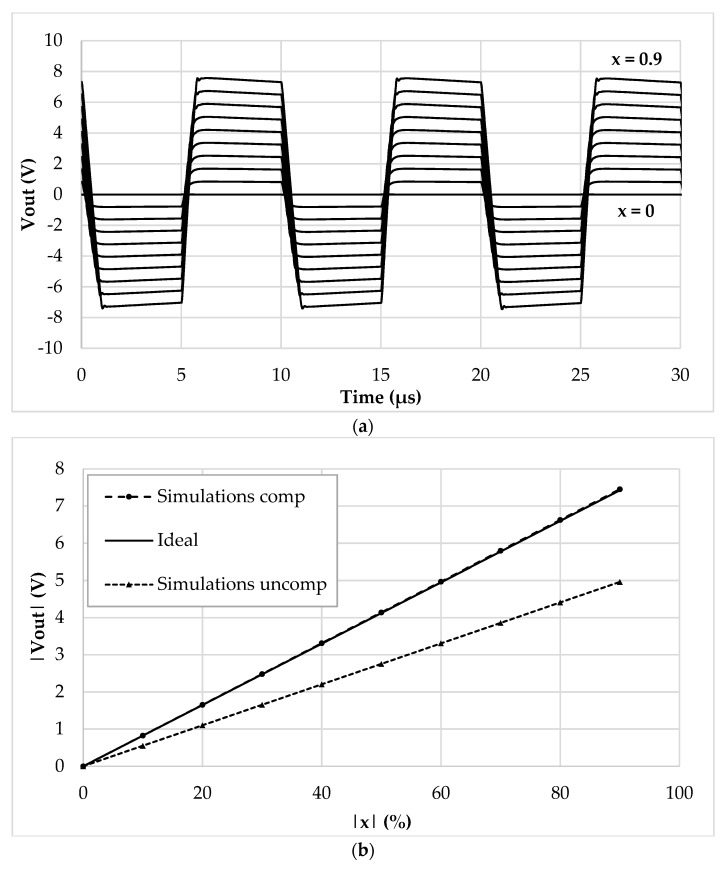

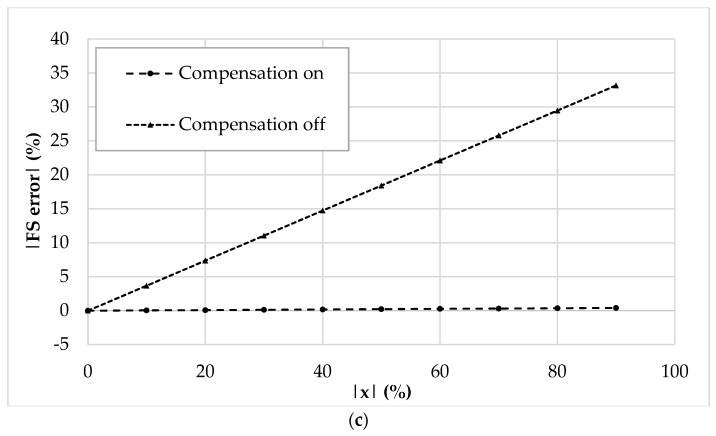
Discrete interface simulations: (**a**) Time domain output voltage, (**b**) extracted DC characteristic, (**c**) full-scale error (%).

Sensor baseline *C_bl_* and parasitic capacitance *C_p_* have been set to 200 pF and 120 pF, respectively. The reference current *I_ref_* has been set to 250 µA peak amplitude and 100 kHz frequency. Figure 9 acknowledges the functionality of the discrete interface and of the compensation mechanism, showing the time domain behavior (Figure 9a), the DC characteristic (Figure 9b), and the full-scale error (Figure 9c).

**Figure 10:**
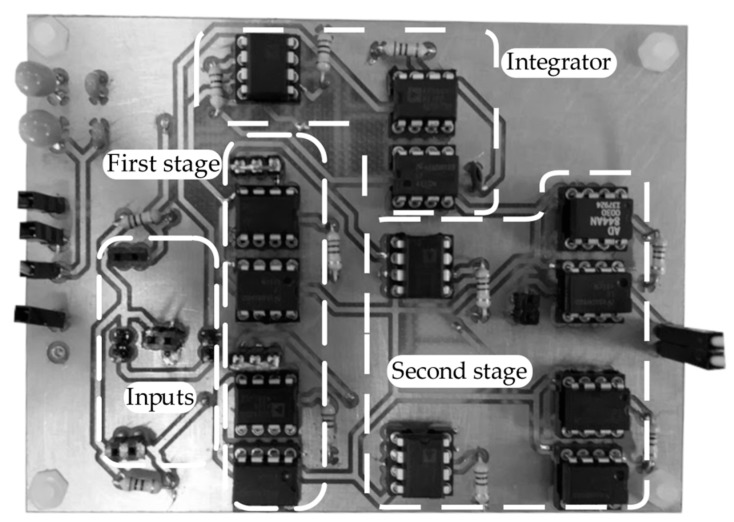
The prototyped interface.

**Figure 11:**
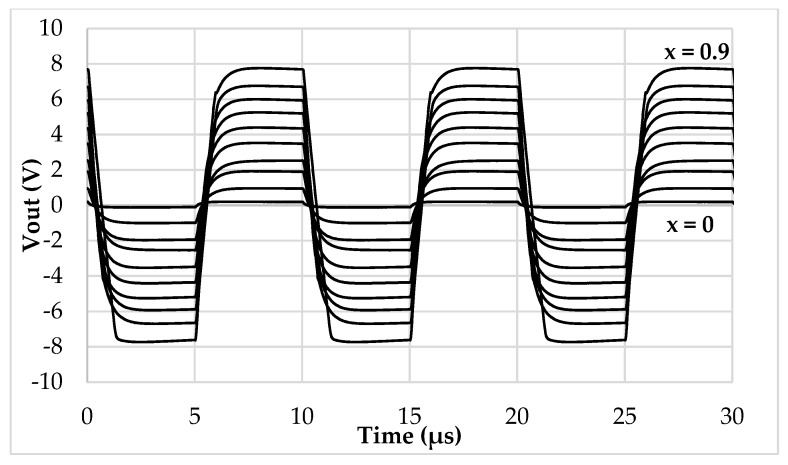
Time domain output voltage of the prototyped interface.

**Figure 12:**
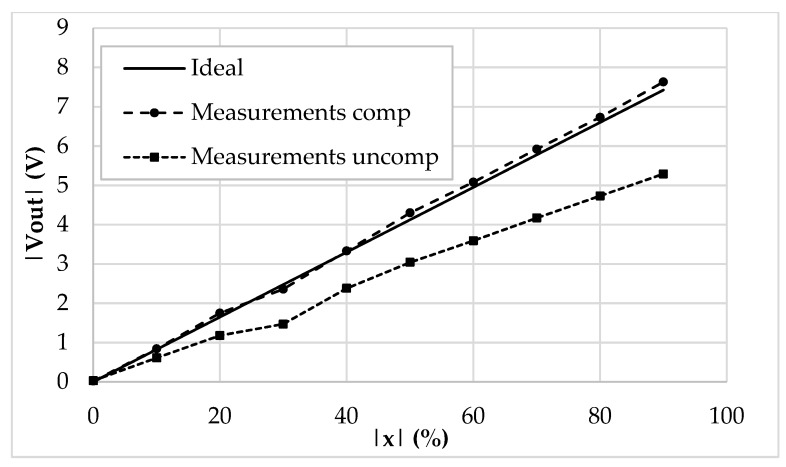
DC characteristic (|*V_out_*| vs. |*x*|) of the prototyped interface.

**Figure 13:**
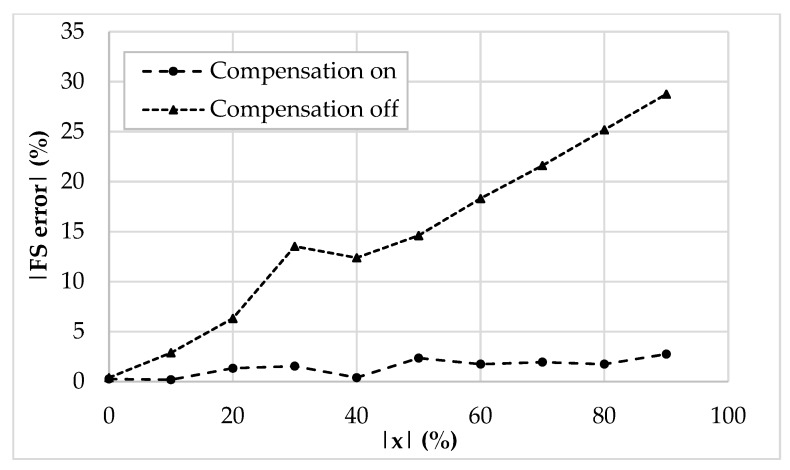
Full-scale error (%) of the compensated and uncompensated prototyped interface.

In Figure 10, the prototyped board is shown. All the parameters have been set to match the ones used in the simulation environment. The reference current has been generated through a Keysight 33600A signal generator, which, together with two resistors and two current buffers, has been able to output accurate values of 250 µA and 500 µA for *I_ref_* and 2*I_ref_*. The output voltage has been measured through a Keysight MSOX3054T oscilloscope and a Keysight N2843A probe. To emulate the behavior of a 200 pF baseline linear differential capacitive sensor (Figure 1b), a set of reference capacitors have been used. The test bench allows us to freely add parasitic capacitances as in Figure 2, so as to mimic even harsh conditions when stray capacitances are comparable to the baseline of the sensor. The time domain measurements are reported in Figure 11, while Figure 12 shows the DC characteristic of the prototype board. As visible, the behavior of the interface is very close to the simulations and the full-scale error (Figure 13) remains always below the 2.5%. The maximum linearity error is equal to 0.9%FS. The presented setup allows us to obtain a sensitivity of 21 mV/pF.

**Figure 14:**
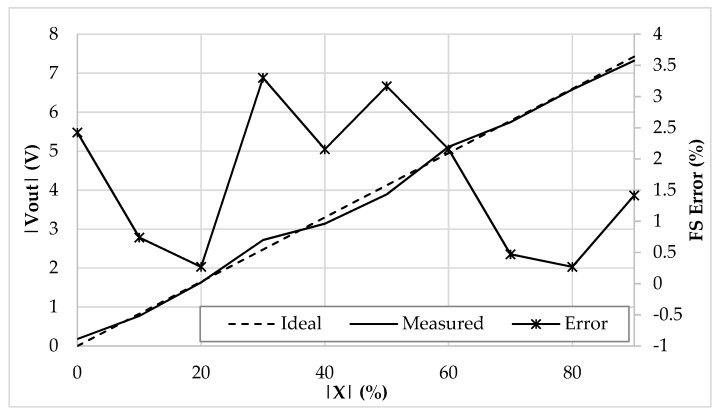
DC characteristic (|*V_out_*| vs. |*x*|) and full-scale error (%) of the prototyped interface with a 10 pF sensor baseline.

Although, without reference capacitors, the interface capability of reading very low baseline values has been investigated as well. A set of 1% tolerance capacitors was adopted to mimic a 10 pF baseline sensor. The DC characteristic is shown in Figure 14: As expected, the error increases up to the 3.3%, however, the interface still shows good behavior when the maximum linearity error is equal to 1.9%FS. The resulted sensitivity is equal to 412 mV/pF. The maximum value of *C*_0_ i.e., *C*_0*max*_ is 200 pF, and its minimum value *C*_0*min*_ is 10 pF. Therefore, the dynamic range is *C*_0*max*_ − *C*_0*min*_ = 200 pF − 10 pF = 190 pF.

Table 2 compares the presented readout technique with data available from the literature: As visible, the advantages coming from a mixed current–voltage approach, together with the consequent ease in alleviating the effects of parasitic capacitances, allow us to maintain a full-range readout capability if compared to traditional voltage mode circuits, while sensibly reducing the allowable sensors baseline. The sensitivity parameter reported in the table is calculated from the output voltage relationships of the referenced works and refers to the minimum baseline allowed by the interface. Since the circuit in [21] has a nonlinear input–output relationship, the sensitivity cannot be uniquely determined.

## 5. Conclusions

A parasitic insensitive readout circuit for differential capacitive sensors has been presented. The proposed circuit utilizes VCII and produces an output voltage proportional to the sensor variation. The working principle is based on the current summing and subtracting technique, resulting in a very simple yet effective implementation. To reduce the errors to the minimum value, no switches are employed. The effect of parasitic capacitances is cancelled using a very simple compensation circuit. The proposed method has been theoretically analyzed and simulated. The simulation results have shown a negligible full-scale error, even for parasitic capacitances comparable to the baseline value. The notable advantage of the proposed circuit is that the sensitivity can be simply adjusted by a resistor. Measurement collected from a discrete prototype has been given, proving good agreement with theory and simulation results and the capability of the circuit to host low baseline sensors. These results confirm the good potentiality of the proposed topology and all the benefits that could come from the on-chip measurements. Several degrees of freedom are also available for the designer to better optimize the performances to her/his necessity. A comparison with the available literature has also been proposed, highlighting the effectiveness of the presented idea and the good potential of the integrated version.

## Figures and Tables

**Table 1:** The used VCII parameters.

Parameter		Value
*r_x_*		800 kΩ
*r_y_*		50 Ω
*r_z_*		80 Ω
*α*		−43 mdB
*β*		−45 mdB
Voltage noise density at	*x*	342 nV/√Hz
	*y*	420 nV/√Hz
	*z*	118 nV/√Hz
Power dissipation		0.7 mW

**Table 2:** Comparison table.

Ref.	This Work	[8]	[10]	[12]	[16]	[17]	[21]
Approach	Mixed	C to V	C to I	C to V	C to V	C to Digital	C to V
Variation Range	±100%	±100%	±100%	±60%	±50%	±50%	−30%; +100%
*C_bl_*	10 pF/200 pF	140 pF ÷ 14 nF	1 pF	20 pF	500 pF	400 pF	400 pF
Linearity Error FS	<1.9%/<0.9%	0.5 ÷ 0.8%	±1.5%	<0.1%	<0.03%	<0.2%	<0.45%
Sensitivity	412 mV/pF/21 mV/pF	71 mV/pF	50 nA/fF	833 mV/pf	5 mV/pF	4 counts/pF	Nonlinear
Typology	Discrete	Discrete	Integrated	Discrete	Discrete	Discrete	Discrete
